# Going web or staying paper? The use of web-surveys among older people

**DOI:** 10.1186/s12874-020-01138-0

**Published:** 2020-10-08

**Authors:** Susanne Kelfve, Marie Kivi, Boo Johansson, Magnus Lindwall

**Affiliations:** 1 Division Ageing and Social Change (ASC), Department of Culture and Society (IKOS), Linköping University, SE-581 83 Linköping, Sweden; 2 Aging Research Center (ARC), Karolinska Institutet & Stockholm University, Tomtebodavägen 18A, SE-171 65 Solna, Sweden; 3 Department of Psychology, University of Gothenburg, Box 100, SE-405 30 Göteborg, Sweden; 4 The Swedish School of Sport and Health Sciences (GIH), Box 5626, SE-114 86 Stockholm, Sweden

**Keywords:** Survey mode, Older adults, Web-survey, Web-push methodology, Non-response, Generalizability, Retirement, Sociodemographic differences

## Abstract

**Background:**

Web-surveys are increasingly used in population studies. Yet, web-surveys targeting older individuals are still uncommon for various reasons. However, with younger cohorts approaching older age, the potentials for web-surveys among older people might be improved. In this study, we investigated response patterns in a web-survey targeting older adults and the potential importance of offering a paper-questionnaire as an alternative to the web-questionnaire.

**Methods:**

We analyzed data from three waves of a retirement study, in which a web-push methodology was used and a paper questionnaire was offered as an alternative to the web questionnaire in the last reminder. We mapped the response patterns, compared web- and paper respondents and compared different key outcomes resulting from the sample with and without the paper respondents, both at baseline and after two follow-ups.

**Results:**

Paper-respondents, that is, those that did not answer until they got a paper questionnaire with the last reminder, were more likely to be female, retired, single, and to report a lower level of education, higher levels of depression and lower self-reported health, compared to web-respondents. The association between retirement status and depression was only present among web-respondents. The differences between web and paper respondents were stronger in the longitudinal sample (after two follow-ups) than at baseline.

**Conclusions:**

We conclude that a web-survey might be a feasible and good alternative in surveys targeting people in the retirement age range. However, without offering a paper-questionnaire, a small but important group will likely be missing with potential biased estimates as the result.

## Background

A web-based survey offers a cheap and convenient mode of data collection, but require that people in the target group have access to internet as well as being willing to answer a web-survey [1–3]. In this paper, we are interested in the potential use of web-surveys among older people. Our rationale is that internet use are more common in cohorts approaching old age today, than in older cohorts [4]. So far, there is limited research on the use of web-based surveys targeting older people and its potentials.

Traditionally, survey data has been collected by interviews (face-to-face or telephone) or by paper questionnaires, usually sent by post. New technologies, such as internet and common access and use of computers and smart phones, provide us with new and efficient possibilities to collect survey data. During the last decades, there has been a dramatic increase in the use of web-surveys, used either as an alternative or as a complement to a postal questionnaire or as a stand-alone tool, where a web questionnaire is the only alternative [2, 5–7]. Since population surveys usually lack a sample frame including email addresses to the population, a web-push methodology is often used, were the survey invitation is sent by mail with a link to the web-survey, with the offer of a paper questionnaire only introduced at a later stage, such as in the last reminder [8, 9].

At the same time as this new technology develops and makes it easier and cheaper to conduct a survey, survey research is suffering from decreasing response rates; a trend that has developed over several decades [10, 11]. Much effort is usually required to reach an acceptable response rate and to achieve a study sample that is generalizable to the population it is supposed to represent [7, 12]. As such, researchers face the challenges of choosing the most efficient survey strategy to the most reasonable cost.

The best choice of survey mode (interview, paper, web or a mixed-mode) depends on the purpose of the study, the target population, and financial resources. The main advantages of web-surveys are their low cost and potential quick access to data and their allowance of respondents to complete the questionnaire whenever and wherever they prefer with the use of different platforms, such as computer or mobile devices [2, 3]. The obvious drawback is that not all people have access to or are familiar with the use of the internet. In general, young people are more frequent users than older people. Hence, a web questionnaire might be more efficient than a paper questionnaire in younger age groups, due to a more widespread use of and access to the internet, whilst it might be more challenging among older individuals [3, 6, 13].

However, the use of internet is also increasing among older people. According to Statistic Sweden, 94% of people 55–64 years old in Sweden reported in 2018 that they have access to internet in their home. Corresponding numbers for people 65–74 and 75–85 years old are 86 and 68%, respectively. In the age group 55–64 years old, 85% report that they use the internet more or less every day. Among people 65–74 and 75–85 years old, corresponding numbers are 69 and 42% [4]. Hence, it might be possible to use a web-survey also among older people, at least among the young olds.

Internet use is also associated with several other sociodemographic factors, besides age. Less internet use has been associated with lower education and female gender [14, 15], as well as lower level of resources, such as income, education and social contacts among older people [16]. Research also found that non-use of internet is associated with lower education, unemployment, disability, and social isolation and that these associations has become stronger over time. Accordingly, the group of people who does not use the internet has become a more vulnerable group over time. In addition, today, non-users often report non-interest as the reason for the non-use, not lack of access to the internet [17]. It is most likely that this group of non-users would be missing in a web-survey if no other mode of options were offered.

Population-based research are currently shifting towards the use of more web-surveys. The expectation is that previous problems with under-coverage in web-surveys will decrease when internet use is becoming more spread across all societal groups [18]. So far, response-rates have been found to be consistently lower in web-surveys compared with other survey modes [19] and web-surveys have been found to have lower survey representativeness compared with other single mode surveys [5]. The exception is among the younger age groups, were a web-survey is likely to generate similar response rates as a paper-survey [3, 13]. Paper-surveys also obtained higher response rates and a demographically more similar sample compared to surveys using the web-push methodology 10 years ago [20]. But, in more recent studies, conducted among younger adults and in a population with high prevalence of internet use, a web-push approach showed significantly higher response rates compared with a traditional paper-survey [8, 9].

Few studies have so far investigated the potential of using the web as a survey mode among older people. There are evidence supporting a mixed-mode approach (web and paper) in surveys of older people, although most older adults still seem to prefer a paper questionnaire [21]. There is also mixed evidence whether web-respondents differ substantially from paper-respondents [20, 22] or not [21].

With this paper, we aim to increase the knowledge about the potentials of using web-surveys among older people. We did this by investigating response patterns and outcomes in an already existing longitudinal retirement study in which a web-push methodology was used and a paper questionnaire was offered as an alternative to the web-questionnaire in the last reminder. Our specific research questions were a) if response patterns (web or paper) differed by sociodemographic factors, self-rated health and psychological outcomes, and b) if results from the study would have varied systematically depending on survey design (i.e., whether or not paper was offered as an alternative to web or not).

## Methods

### Data material

We used three waves of data from the Health, Ageing and Retirement Transitions in Sweden study (HEARTS), a survey based on a nationally representative sample of the Swedish population, 60–66 years old at baseline 2015, with yearly follow-ups [23]. The sample was generated as a probability sample from the National Register on the Total Population, covering all inhabitants registered in Sweden, by Statistic Sweden. Data is primarily collected using a web-questionnaire, but a paper-questionnaire is offered as a choice in the final reminder letter. The questionnaire contains questions largely focusing on various aspects of health and well-being and in relation to retirement, besides questions about sociodemographic factors. The time needed to respond to all questions varies considerably between individuals, but is typically in the range of 50–90 min, and no incentives are used.

**Fig. 1:**
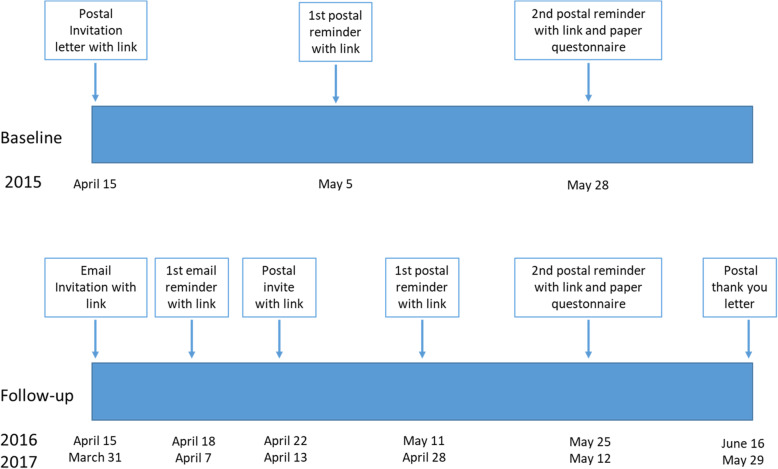
Timeline for data collection in the Health, Ageing and Retirement Transitions in Sweden study (HEARTS)

At baseline, an invitation letter to the study was distributed by postal mail, including a link to the web-questionnaire. Two reminders were sent by post, in which the last reminder also included the full paper-questionnaire (Fig. 1). For follow-ups, the first invitation, as well as the first reminder, was sent by email to those who previously gave their email address. Thereafter, a postal invitation was sent to all people that did not responded to the email invitation (including those who did not gave an email address), as well as two postal reminders in which the last reminder included the full paper-questionnaire, in line with the data collection strategy at baseline. Finally, a last postal reminder, including a thank you for participating in previous waves, was sent. The present study is based on data from the three first waves of the HEARTS study.

### Background measures

*Sex* and *age* was registered during the sampling procedure. *Education* was measured by self-reported highest level of *education* (coded into; primary or below, secondary, or tertiary education). We also used self-reported *country of birth* (Sweden or outside Sweden), self-reported *marital status* (married/partner, unmarried, divorced/separated, widow/widower), and self-reported *retirement status* (not retired, retired and working-consider myself a worker, retired and working-consider myself a retiree, fully retired).

### Outcome measures

To evaluate the effect of survey design, we chose three different key outcome measures; *depression* measured by CES-D scale [24], *life-satisfaction*, measured by Diener’s 5 item scale [25], and *self-rated health*, measured by the question “How is your general health”. The answers “Very bad”, “Bad” or “Poor” was coded into *poor self-rated health*, and “Fair”, “Good” or “Very good” into *not poor self-rated health*.

### Sample groups

To answer the research questions we created two sets of sample groups:

1a) Baseline web-sample (those who answered by web at baseline).

1b) Baseline paper-sample (those answering by paper at baseline).

2a) Longitudinal web-sample (people that answered by web in all three waves),

2b) Longitudinal mix-sample (those switching between paper and web between the three waves).

2c) Longitudinal paper-sample (those answering by paper in all three waves).

The differences between the first and the second set of sample groups is that the longitudinal sample groups (2a, 2b and 2c) are restricted to people who answered all three waves. Hence, the longitudinal sample groups used in this study do not include people with any non-response, although non-respondents, except for the baseline non-responders, are invited to participate in subsequent waves.

### Analyses

First, we created a flow chart of the response patterns, to examine how people moved between web-response, paper-response, and non-response across the three waves. Second, we compared the sample groups 1a and 1b, as well as 2a, 2b and 2c, regarding background factors and the three specific outcome indicators (i.e. depression, life satisfaction, and self-rated health). In the analyses, we used the Chi2-test or t-test, depending on outcome measure.

Finally, we compared the association between retirement status and the three outcome measures in the different sample groups, separately and together, to analyze the effect of offering the paper questionnaire as an alternative. The underlying assumption is that the alternative to a paper response would have been a complete non-response. Since retirement status was a grouping variable in these last analyses, we restricted the sample in these analyses to those who were either “not retired” or “fully retired”, and excluded those with less clear retirement status, who stated that they were “retired and working-consider myself a worker” (*n* = 443 at baseline, *n* = 507 at 2nd follow-up) or “retired and working-consider myself a retiree” (*n* = 260 at baseline, *n* = 402 at 2nd follow-up) or had missing information (*n* = 155 at baseline, *n* = 14 at 2nd follow-up). The results for continuous outcomes are based on linear regressions and presented as unstandardized beta-coefficients (β). Results for binary outcomes were calculated by logistic regressions but presented as Average Marginal Effects (AMEs), due to the problem of comparing odds ratios over different models based on different groups [26]. The AME gives the predicted absolute differences in proportion from the reference category, given the same value in all other variables included in the model. All models were calculated crude as well as adjusted for sex, age, education, and civil status. Analyses were performed using STATA 14.

## Results

**Fig. 2:**
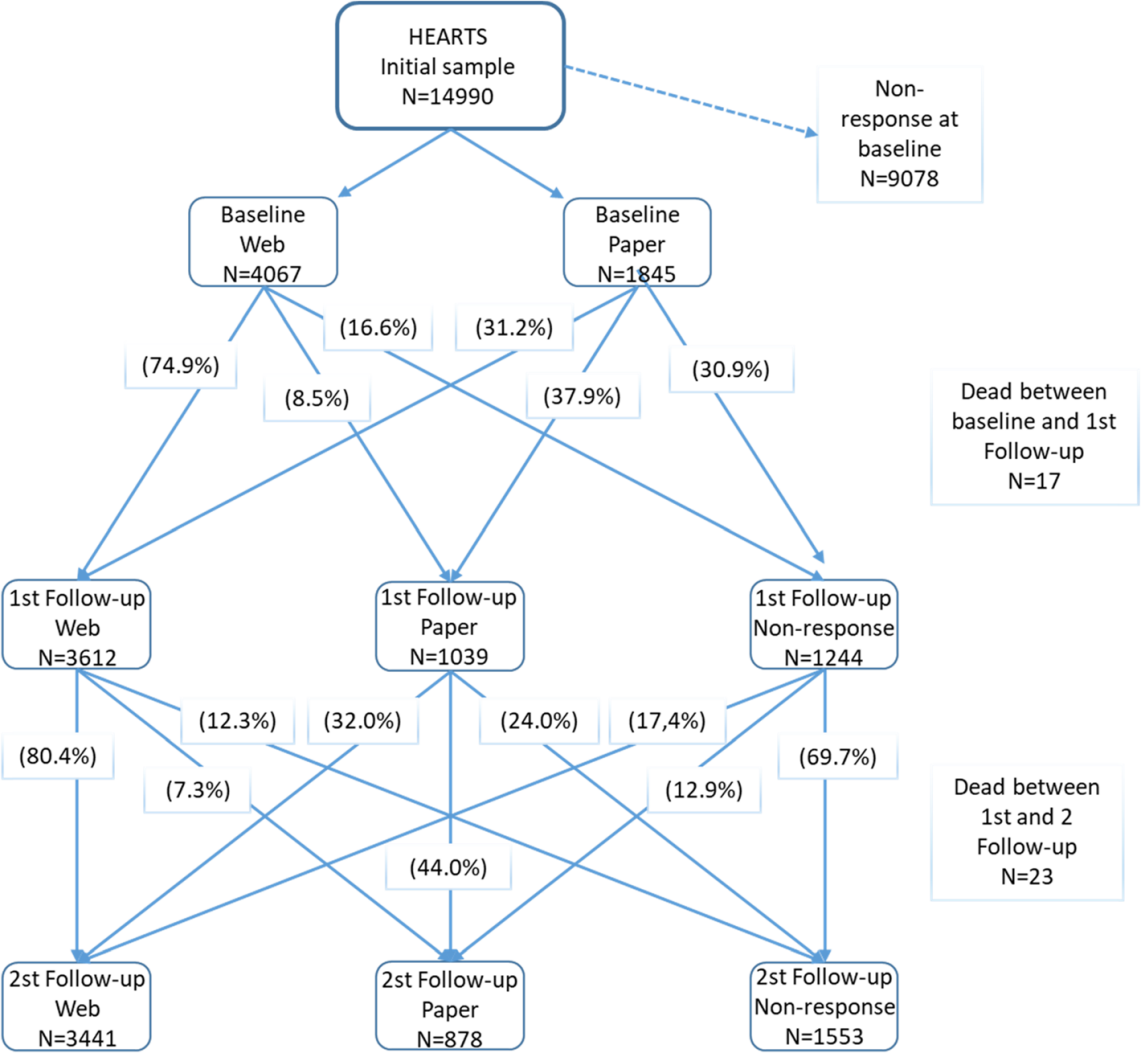
Flow chart of the response patterns in the Health, Ageing and Retirement Transitions in Sweden study (HEARTS)

Out of the total sample (*n* = 14,990), 39.4% (*n* = 5913) answered the questionnaire at baseline, 27.1% (*n* = 4067) by web and 12.3% (*n* = 1845) by paper (Fig. 2). Among this baseline response group, 78.7% also answered the 1st follow-up and 73.1% the 2nd follow-up. In total, 40 baseline responders died between baseline and 2nd follow-up (17 before 1st follow-up and 23 between 1st and 2nd follow-up).

The response patterns illustrated in Fig. 2 reveal four main findings. First, at all three waves, a majority of the respondents answered by web. Second, a majority of the web-respondents answered by web also in subsequent wave (74.9 and 80.4% for the1^st^ and 2nd follow-up respectively). In contrast, people that did not respond until they got a paper questionnaire, i.e. paper-respondents, were less stable in their preference over waves, that is, they were more evenly distributed between web and paper response in subsequent wave. Third, paper-respondents were twice as likely to be non-respondent in the subsequent wave compared to web-respondents (30.9% vs 16.6%; *p* < 0.001 between baseline and 1st follow-up and 24.0% vs 12.3%; *p* < 0.001 between 1st and 2nd follow-up). Fourth, 30.3% of the non-responders at 1st follow-up did a re-entry into the study at the 2nd follow-up.

In total, 42.7% (*n* = 2510) of the baseline sample still alive at 2nd follow-up (*n* = 5873) answered by web at all three waves, 6.2% (*n* = 369) answered by paper at all three waves, 18.1% (*n* = 1065) switched modes between waves and 32.8% (*n* = 1929) did not respond to all three waves.

We found significant socio-demographic differences between web and paper respondents (Table 1). Compared with web-respondents, paper-respondents at baseline were more likely to be women (59.2% vs 51.4%; *p* < 0.001), have lower education (23.5% vs 12.1% with primary education and 39.4% vs 55.3% with tertiary education; *p* < 0.001), born outside Sweden (13.2% vs 10.7%; *p* = 0.008), fully retired (25.3% vs 20.5%; *p* < 0.001), and less likely to be married (68.4% vs 75.1%; *p* < 0.001).

**Table 1:** Sociodemographic differences between the sample groups (for baseline samples: web and paper respondents; for longitudinal samples: web, mix and paper respondents among those that answered all three waves)

Characteristics measured at baseline	HEARTS baseline web sample (*n* = 4068)	HEARTS baseline paper sample (*n* = 1845)	*P*-value	HEARTS longitudinal web sample (*n* = 2510)	HEARTS longitudinal mix sample (*n* = 1065)	*P*-value	HEARTS longitudinal paper sample (*n* = 369)	*P*-value
*Age*								
Mean age	63.1	63.2	*0.075*	63.1	63.3	*0.011*	63.5	*< 0.001*
*Sex*								
Women	51.4	59.2	*< 0.001*	50.6	60.4	*< 0.001*	63.5	*< 0.001*
*Education*								
Primary or below	12.1	23.5		10.5	14.5		28.0	
Secondary	32.7	37.2		31.9	32.9		40.4	
Tertiary	55.3	39.4	*< 0.001*	57.7	52.6	*0.001*	31.6	*< 0.001*
*Marital status*								
Married/partner	75.1	68.4	*< 0.001*	77.4	75.2	*0.313*	57.5	*< 0.001*
*Country of birth*								
Other than Sweden	10.7	13.2	*0.008*	9.1	8.2	*0.414*	10.8	*0.290*
*Retirement status*								
Fully retired	20.5	25.3	*< 0.001*	20.9	23.1	*0.159*	32.9	*< 0.001*

Note: Age is presented as mean values with p-values from t-tests. All other variables are presented as proportions with *p*-values from Chi2-tests. *P*-values refers to comparison with the baseline or longitudinal web-sample

These differences were compounded in the longitudinal sample groups. That is, when comparing those who answered by web across the three waves with those answering by paper in all three waves, the differences between web and paper response groups were more pronounced for all socio-demographic factors, such as 32.9% vs 20.9% (*p* < 0.001) fully retired among the longitudinal paper-sample compared with the longitudinal web-sample. The estimates for the response group that switched mode between waves, that is, the longitudinal mix-sample, were placed between the estimates from the longitudinal web- and paper-samples, indicating a dose response relationship between the sample groups and the baseline characteristics.

The only exception was country of birth. The proportion of people born outside Sweden was lower in the longitudinal sample compared with the baseline sample, both among web-respondents and paper-respondents and the significant differences between web and paper respondents that were present at baseline disappeared in the longitudinal sample. Age seems to have a minor impact on response mode, although the mean age among paper-respondents and mix-respondents was slightly higher than among web-respondents in the longitudinal sample groups.

Paper-respondents reported higher mean level of depression (4.4 vs 4.0; *p* = 0.003) as well as lower self-rated health (15.7% vs 9.9% with poor self-rated health; *p* < 0.001) at baseline (Table 2). These differences were also compounded in the longitudinal sample groups (4.0 and 5.0 vs 3.5 for mean value of depression; *p* = 0.001 and *p* < 0.001 and 10.6 and 19.3% vs 8.5% for proportion of poor self-rated health; *p* = 0.050 and *p* < 0.001). No significant differences in life satisfaction was found between web and paper respondents at baseline, but mix and paper respondents reported slightly lower mean level of life satisfaction than web respondents in the longitudinal sample groups (24.3 and 23.3 vs 24.8; *p* = 0.039 and *p* < 0.001).

**Table 2:** Differences in depression, life satisfaction and poor self-rated health between the sample groups (measured at baseline for baseline web and paper sample and at 2nd follow-up for the longitudinal web, mix and paper sample)

Outcomes measured at baseline and at 2nd follow-up	HEARTS baseline web sample (*n* = 4068)	HEARTS baseline paper sample (*n* = 1845)	*P-value*	HEARTS longitudinal web sample (*n* = 2510)	HEARTS longitudinal mix sample (*n* = 1065)	*P-value*	HEARTS longitudinal paper sample (*n* = 369)	*P-value*
*Depression scale (0-30p*								
Mean	4.0	4.4	*0.003*	3.5	4.0	*0.001*	5.0	*< 0.001*
*Life satisfaction scale (7-35p)*								
Mean	24.2	24.1	*0.781*	24.8	24.3	*0.039*	23.3	*0.001*
*Poor self-rated health*								
%	9.9	15.7	*< 0.001*	8.5	10.6	*0.050*	19.3	*< 0.001*

Note: Estimates of Depression and Life satisfaction are presented as mean values with p-values from t-tests. Poor self-rated health are presented as proportions with *p*-values from Chi2-tests

Finally, we found that the association between retirement status and depression, as well as self-rated health, differed by sample group. Retired people reported lower level of depression at baseline (β = − 0.65; *p* < 0.001) compared with non-retired people (Third column in Table 3). However, dividing the sample by response group (first and second column) revealed that the association between retirement status and depression was only present among web-respondents (β = − 0.89; *p* < 0.001 vs β = − 0.22; *p* = 0.378 in the paper sample). We found the same pattern in the longitudinal sample; retired people reported lower level of depression at 2nd follow-up (β = − 0.73; *p* < 0.001), but only among people in the web-sample (β = − 0.87; *p* < 0.001) and the mix-sample (β = − 0.86; *p* = 0.006), not in the paper sample (β = 0.02; *p* = 0.980).

**Table 3:** Depression, Life Satisfaction and Poor Self-rated health among retired people compared with non-retired people, by sample group

	HEARTS baseline web sample (*n* = 3491)		HEARTS baseline paper sample (*n* = 1564)		HEARTS baseline total sample (*n* = 5055)		HEARTS longitudinal web sample (*n* = 1962)		HEARTS longitudinal mix sample (*n* = 821)		HEARTS longitudinal paper sample (*n* = 303)		HEARTS longitudinal total sample (*n* = 3086)	
	β^*a*^	*P-value*	β	*P-value*	β	*P-value*	β	*P-value*	β	*P-value*	β	*P-value*	β	*P-value*
*Depression scale (0-30p)*														
Crude model	−0.89	*< 0.001*	−0.22	*0.378*	−0.65	*< 0.001*	−0.87	*< 0.001*	−0.86	*0.006*	0.02	*0.980*	−0.73	*< 0.001*
Adjusted model^c^	−0.36	*0.080*	−0.17	*0.608*	−0.19	*0.272*	−0.81	*0.001*	−1.13	*0.011*	−0.34	*0.741*	−0.87	*< 0.001*
*Life satisfaction scale (7-35p)*														
Crude model	1.76	*< 0.001*	1.74	*< 0.001*	1.75	*< 0.001*	2.04	*< 0.001*	1.36	*0.008*	2.45	*0.014*	1.83	*< 0.001*
Adjusted model^c^	0.90	*0.007*	1.58	*0.004*	1.13	*< 0.001*	2.03	*< 0.001*	2.05	*0.004*	2.64	*0.109*	2.09	*< 0.001*
	*AME*^*b*^	*P-value*	*AME*	*P-value*	*AME*	*P-value*	*AME*	*P-value*	*AME*	*P-value*	*AME*	*P-value*	*AME*	*P-value*
*Poor Self rated health*														
Crude model	−0.86	*0.477*	1.39	*0.497*	0.20	*0.854*	−3.86	*0.003*	0.86	*0.710*	0.10	*0.983*	−1.89	*0.094*
Adjusted model^c^	1.70	*0.305*	5.01	*0.090*	2.93	*0.047*	−3.68	*0.045*	−1.76	*0.595*	−8.39	*0.338*	−3.35	*0.043*

^a^Unstandardized beta coefficient

^b^Average Marginal Effect, interpreted as the estimated absolute differences in proportion with Poor Self-rated Health among retired compared with non-retired people

^c^Adjusted for sex, age, education and civil status

Retired people also reported better self-rated health (lower proportion of poor self-rated health) compared with non-retired people in the longitudinal sample, an association that we only found among web-respondents (AME = -3.86; *p* = 0.003 vs AME = 0.86; *p* = 0.710 in the mix-sample and AME = 0.10; *p* = 0.983 in the paper-sample). No significant differences in self-rated health by retirement status was observed at baseline.

However, when adjusting the models for age, sex, education and civil status, no clear differences emerged between web and paper respondents regarding the association between retirement status and depression and self-rated health.

All sample groups showed a similar pattern regarding the association between retirement and life satisfaction. Retired people reported better life satisfaction compared with non-retired, both among web respondents (β = 1.76; *p* < 0.001 at baseline and β = 2.04; *p* < 0.001 at the 2nd follow-up), paper respondents (β = 1.74; *p* < 0.001 at baseline and β = 2.45; *p* = 0.014 at the 2nd follow-up) and among respondents that switched mode between waves (β = 1.36; *p* = 0.008).

## Discussion

In this study, we investigated response patterns in the Swedish HEARTS study on retirement transition, in which a web-push methodology was used with paper questionnaires offered as an alternative to the web questionnaire in the last reminder.

Our results can be generalized into three main findings. First, most respondents answered by web (69%; *n* = 4067 at baseline) and this was a rather stable group who continued to respond by web in subsequent waves. Paper-respondents on the other hand, that is, those who did not respond until they got a paper questionnaire with the last reminder, were fewer (31%; *n* = 1845 at baseline) and were less stable in subsequent waves, with higher probability of non-response and changing response mode.

Second, compared with those who answered by web only, paper-respondents, as well as those that switched modes between waves, were more likely to be women, have a low level of education, being non-married and fully retired and reported more depression and poor self-rated health. In addition, the associations between retirement status and depression and to some extent poor self-rated health were stronger among web-respondents than among paper-respondents, although this result must be interpreted with caution, since some of these estimates changed substantially when adjusting for confounders.

Third, the differences between web and paper respondents were more pronounced in the longitudinal sample, compared with the baseline sample; the differences between web and paper respondents increased when taking into account the longitudinal response patterns, that is, dividing the longitudinal sample by those that answered by paper across all three waves, those that switched mode between waves and those who responded by web across all three waves.

Notable is also that the response rate in the HEARTS study is similar [9] or even higher [8] than recent studies using web-push methodology among younger age groups. This indicates that a web-push methodology may be efficient when collecting survey data among older adults, at least in countries with widespread internet use.

Our results are in line with previous studies of surveys of older individuals, where women and non-married [22], low educated [21, 22] and non-working people [21] were found to be less likely to answer a web-questionnaire. Our finding that people answering by web had better subjective health are both supported [22] and non-supported [21] by previous studies. However, different measures of health were used in the compared studies.

Our finding that the response rate in the subsequent wave was higher among web-respondents than among paper-respondents are also in line with the previous literature. It has been shown that the differences in response rate between paper and web surveys is lower among panel members than among one-time respondents [7]. This suggest that given survey response by web one time, the likelihood of response to the next wave of a web-survey are higher than in a new sample where people are contacted for the first time. On the other hand, it should be mentioned that in the HEARTS study, the paper option was only offered in the last reminder. Hence, the paper-respondents in HEARTS are not comparable with paper respondents from a survey with a paper option in the first invitation. It is likely that some people from the web-sample would have preferred the paper version if they had the choice, without being less likely to participate in the subsequent wave. These results imply that the group of respondents that did not answer until they got a paper questionnaire are also the people that are most likely to not participate in a survey. It should also be mentioned that those respondents that once answered by paper might be less likely to answer by web in subsequent waves, as they know about the coming paper option.

From a previous study, we know that the attrition in the HEARTS study is associated with personality; people with higher scores on extraversion and neuroticism, and lower scores on agreeableness, were more likely to drop out [27]. Results from the present study adds to that knowledge by showing that those who did not answer until they got a paper questionnaire, that is, the paper respondents, were also more likely to attrite from the study. Finally, our analyses also showed that differences between the longitudinal sample groups (web vs paper respondents) were greater than between web and paper respondents at baseline. This finding demonstrates that without the option of a paper questionnaire, the response group in HEARTS would have been even more selected over time if not a paper questionnaire would have been offered.

### Implications of the chosen survey design

It is not possible to estimate what the response rate in the HEARTS study would have been if data had been gathered using another survey mode. Previous studies show that web surveys in general produce approximately 10–11% lower response rate than other survey modes, such as paper and telephone [7, 28]. A recent meta-analyses, including over 100 experiments, confirm these results and show a 12% response rate difference between web-surveys and other modes [19]. The exception is among students, were the results are more mixed. In one study among students, paper and web yielded the same response rate [3], but in another study the highest response rate was reached when both paper and web was offered [29]. Further, in an experimental study of a highly internet-literate population, the offer of both web and paper did not improve response rate compared to only paper. Nevertheless, offering paper at a later stage, as an alternative to web, improved the response rate and was equivalent to the use of paper as the only alternative [30]. Previous research also show that the number of reminders seems to be less efficient in web surveys than in other modes, such as paper [7, 13]. Taken together, this implies that it is likely that the non-response rate in HEARTS would have been higher if not a paper questionnaire was offered as a response option, even if more reminders would have been used.

The major problem with non-response, in addition to the decreased statistical power, is largely related to the risk that the non-response is occurring non-random. Web and paper respondents differed significantly from each other in the HEARTS study, not only in sociodemographic factors, but also in self-reported health and certain psychological outcomes, both in levels and regarding the association with retirement status. In addition, these differences was compounded in the longitudinal sample. That is, the differences between web and paper respondents was more substantial in the longitudinal sample (i.e. among those who either answered by web or by paper across all three waves) compared with all those answering at baseline. Hence, we also conclude that without offering a paper questionnaire as an alternative, a small but important group would have been missing in subsequent waves in the HEARTS study.

The next question is whether the quality of the data in HEARTS depended on choice of survey design. We know that survey mode matter for the results and that it can be problematic to change survey mode across waves [31, 32]. However, we also know that some of the differences between survey modes can be explained by changes in wording, structure and visual effect used in the different survey modes and it is therefore recommended to use questions as similar as possible when using multi-mode surveys [32]. In the HEARTS study, wording and structure were as identical as possible in the paper and web questionnaire. In addition, the paper and web questionnaire were self-administered, which implies smaller differences than if one of the modes were self-administrated questionnaires and one was conducted by interview [31]. Further, in a more recent paper, web, paper and telephone mode yielded similar results regarding political opinion and issues [33].

### Limitations

An important limitation in this study is that there is no gold standard to compare our results with, that is, we do not know the real population values for most of the studied variables. However, based on the differences we found between web and paper respondents and that we assume that the offered paper questionnaire contributed with data from a group that otherwise would have been missing, we believe that offering the paper questionnaire generated results closer to the true population values.

This hypothesis is supported by previous research, in which researchers found that a) the use of mail and web modes alone resulted in very different types of respondents, and b) a mix of web and mail obtains respondents quite similar to a mail-only design. From these findings the authors drew the conclusion that the type of people who respond via the web may also respond via mail but not the other way around. Hence, “when offering the web to general public household samples, it is important to provide a mail option to those who cannot or will not respond by Web.” [20].

Compared to other studies that used a design where different groups were offered different options (e.g., web or paper), the design used in the present study, as a part of the general set-up of the HEARTS study, makes it harder to draw clear conclusions in terms of mechanisms underlying differences. For example, the timing of the reply (how long it took for the respondents to reply) is to some extent confounded with the mode of response. One reason the group that answered via web as a reply to the email contact, may be because they prefer email as a contact, or, because they tend to reply to surveys directly instead of waiting. Due to this limitation in the design, we could not in a reliable way distinguish those that responded early via web as a response to the email contact from those that responded somewhat later via web as a response to the mail contact.

Although the HEARTs study is a survey comprising questions of relevance for the target population and therefore might motivate participation, the questionnaire is extensive and rather time-consuming. It is most likely that there are differences between people who felt motivated enough to answer the questionnaire and those who did not. Respondents in HEARTs are for example more educated compared with the general population [23]. It might be that the differences between web and paper respondents, as well as the response patterns, would have been different in a less extensive and time-consuming survey.

## Conclusion

The present study indicate that a web-survey, with a web-push methodology, might be a good and feasible alternative in studying older adults in the pre- and post-retirement ages, i.e. in their 60’s and early 70’s. However, without offering a paper-questionnaire as an alternative to solely a web-questionnaire, a small but important subgroup will be missing which most likely would produce more biased estimates. Our results indicated that without a paper alternative, people with low education, women, fully retired and non-married people would have been underrepresented in the HEARTs study. This would also have resulted in an underestimation of the prevalence of depression and poor self-rated health, whereas the association between retirement and depression would have been overestimated. Notably, we found that the differences between web and paper respondents increased in the longitudinal sample, that is, after two follow-ups, which implies that the potential bias from not providing a paper questionnaire as an alternative would have been even greater in analyses using longitudinal data.

## Data Availability

The data that support the findings of this study are available from University of Gothenburg but restrictions apply to the availability of these data, which were used under license for the current study, and so are not publicly available. Data are however available from the authors upon reasonable request and with permission of University of Gothenburg.

## Abbreviations

HEARTS: The Health, Ageing and Retirement Transitions in Sweden study
AME: Average Marginal Effects
